# An Infant With Hyponatremia, Hyperkalemia, and Faltering Growth: A Case Report

**DOI:** 10.1155/crie/1964629

**Published:** 2026-09-25

**Authors:** Felicia Cooper, Heather Fagan, Sindy Villacres, Pamela Ellsworth, Shilpa Gurnurkar

**Affiliations:** ^1^ Department of Pediatrics, Nemours Children’s Health, Orlando, Florida, USA, nemours.org

## Abstract

Infants presenting with hyponatremia and hyperkalemia routinely engage clinician concern for congenital adrenal hyperplasia (CAH), a set of disorders resulting in difficulty producing glucocorticoids, mineralocorticoids, and sex steroids in the adrenal glands. However, we present the case of an infant with classic CAH‐like symptoms, a negative newborn screen, and clinical features inconsistent with the diagnosis of CAH. Ultimately, the patient was diagnosed with pseudohypoaldosteronism (PHA), a condition in which aldosterone is unable to activate its receptor in the kidney. Notably, he also exhibited an uncommon complication of a left external iliac thrombus. Infants with hyponatremia and hyperkalemia without an immediately identifiable cause should be screened for urinary tract infection (UTI) and urinary tract malformations, and infants with severe urinary tract abnormality or malformation should have electrolytes monitored. This recommendation stems from the increased susceptibility of developing PHA in the setting of UTI without genitourinary tract malformation.

## 1. Introduction

Pseudohypoaldosteronism (PHA) should be considered in the differential diagnosis of infants, especially of the male sex, with poor weight gain, low sodium, and high potassium. Congenital adrenal hyperplasia (CAH) is the most well‐known cause of these specific electrolyte derangements in infancy and must be ruled out. Differentiation between the two conditions is critical, as secondary PHA (sPHA) is treated with fluid resuscitation and antibiotics, with or without sodium supplementation, while CAH is treated with glucocorticoid and mineralocorticoid replacement.

## 2. Case Report

A healthy‐appearing male infant was born to a woman with three living children at 41 weeks of gestation. He had a normal newborn exam, and a weight appropriate for gestational age was noted at birth. Prenatal ultrasounds were reportedly normal, and the infant’s newborn screen demonstrated no abnormalities. There were no diagnosed medical conditions at birth, and he underwent an uncomplicated circumcision. The patient was exclusively breastfed and received 400 units of vitamin D daily. He presented to the pediatrician at 2 months of age with loose stools for the preceding week and a total weight gain of 36 g since birth. Stagnant weight gain was noted at 6 weeks with subsequent weight loss in the following 2 weeks. His measurements at presentation were as follows: weight 3.6 kg (<0.01 percentile), length 55 cm (0.6 percentile), head circumference 37 cm (0.7 percentile), and weight‐for‐length 0.61 percentile (*z* = −2.51). The mother reported breastfeeding every 3 h with a good latch, but the infant seemed routinely fussy and hungry after feeds. Family history was negative for known renal or adrenal disease. The degree of malnutrition noted by the pediatrician was staggering, prompting a referral to the local hospital for evaluation.

At the community hospital emergency department, labs revealed hyponatremia (120 mmol/L) and hyperkalemia (6.9 mmol/L, nonhemolyzed). Serum glucose was normal (80 mg/dL). The infant was transferred to our children’s hospital’s pediatric intensive care unit (PICU) for further evaluation and management, as the outside hospital had limited diagnostic testing and no pediatric subspecialist coverage.

The infant was transported by a critical care transport team, presenting to the PICU in compensated shock with tachycardia and poor perfusion. He received 40 mL/kg of normal saline during transport. In the PICU, he received additional volume resuscitation (30 mL/kg) with isotonic fluids and packed red blood cells. Examination after the initial resuscitation revealed a small‐for‐age infant with decreased muscle mass but an otherwise normal physical exam, including circumcised male genitalia. He was tachycardic to 196 bpm in the absence of fever, with all other vital signs appropriate for age. Intravenous fluids were continued at a maintenance rate, and further evaluation was conducted. Prior to full correction of the metabolic derangements, but after emergent fluid and blood resuscitation, a comprehensive laboratory evaluation was obtained that included endocrine studies (Tables 1 and 2). At that time, serum sodium remained low and potassium was elevated. A high‐dose cosyntropin stimulation test (15 mcg/kg/dose) was performed during this early stabilization phase and demonstrated an appropriate cortisol response (peak >61.6 mcg/dL), effectively excluding adrenal insufficiency from the differential diagnosis. Notably, the patient did not receive any glucocorticoid therapy prior to or during this evaluation. Serum 17‐hydroxyprogesterone and plasma renin levels were normal, ruling out common types of salt‐wasting CAH. An aldosterone level drawn concurrently resulted several days later and was markedly elevated (315.9 ng/dL [ref: 5.0–90 ng/dL]). Hyponatremia was corrected gradually with isotonic fluid resuscitation to mitigate the risk of osmotic demyelination syndrome seen with swift correction of chronic hyponatremia. Serum sodium was measured using direct ion‐selective potentiometry (VITROS System, QuidelOrtho Corporation, San Diego, CA) per institutional laboratory standards. There was no evidence of hyperlipidemia or hyperproteinemia to suggest pseudohyponatremia. Electrolytes were normalized by hospital day 2.

**Table 1:** Notable endocrine laboratory values upon presentation to the pediatric intensive care unit (after fluid resuscitation).

Component (latest ref range)	Value
Sodium (134–142 mmol/L)	133 (L)
Potassium (3.5–5.6 mmol/L)	4.3
Chloride (96–110 mmol/L)	112 (H)
TCO_2_ (17–29 mmol/L)	12 (L)
Anion gap (0–15 mmol/L)	9
Albumin (2.0–4.8 g/dL)	3.6
Glucose (57–117 mg/dL)	120 (H)
T4, free (0.8–2.0 ng/dL)	1.4
T4 total (6.0–14.0 μg/dL)	9.9
TSH (0.50–6.00 mIU/L)	3.55
Cortisol (2.70–18.90 mcg/dL)	51.9
Renin (2.000–37.000 ng/mL/h)	23.962
17‐OH‐progesterone (170 HP ng/dL)	299
ACTH (7.2–63.3 pg/mL)	103.0 (H)
Aldosterone (5.0–90 ng/dL)	315.9 (H)

Abbrevations: ACTH, adrenocorticotropic hormone; H, high; L, low; TSH, thyroid‐stimulating hormone.

**Table 2:** Arterial blood gas results upon presentation to the pediatric intensive care unit.

Component (latest ref range)	Value
pH (7.34–7.43)	7.27 (L)
PCO_2_ (35–45 mmHg)	26 (L)
PO_2_ (83–108 mmHg)	95
HCO_3_ (22–26 mmol/L)	12.0 (L)
TCO_2_, whole blood (24–30 mmol/L)	13 (L)
Base deficit (mmol/L)	15
O_2_ saturation (95%–100%)	96
Sodium, whole blood (134–142 mmol/L)	135
Potassium, whole blood (3.5–5.6 mmol/L)	4.0
Ionized calcium, whole blood (0.97–1.30 mmol/L)	1.53 (H)
Hematocrit (30.5%–37.7%)	24 (L)
Hemoglobin (10.5–13.0 g/dL)	8.2 (L)
Glucose, whole blood 54–117 mg/dL	125 (H)

Abbrevations: H, high; L, low.

During the patient’s evaluation, initial labs also revealed a complete blood count with an elevated white blood cell count (14.5 K/UL) and low hemoglobin (7.8 g/dL). Procalcitonin, C‐reactive protein, prothrombin time (PT), international normalized ratio (INR), and partial thromboplastin time (PTT) were normal. Blood culture revealed no growth for 5 days. The electrocardiogram demonstrated sinus tachycardia, and an echocardiogram revealed normal function and anatomy except for a small patent foramen ovale with a left‐to‐right shunt. Urine studies from a catheterized sample were initially notable for positive nitrites and leukocytes and later grew *Escherichia coli*, consistent with a urinary tract infection (UTI). Complete urine study results are shown in Table 3.

**Table 3:** Urine studies from a catheterized sample upon arrival to the pediatric intensive care unit.

Component (latest reference range/normal)	Value
Color, urine (pale yellow)	Orange
Clarity, urine (clear)	EXCESSIVELY TURBID
Glucose normal (0–15 mg/dL)	Normal
Protein (negative)	1+ (H)
Bilirubin (negative)	Negative
Urobilinogen (normal [<2.0]) mg/dL)	<2.0
pH (5.5–8.5)	5.5
Blood (negative)	2+ (H)
Ketones (negative)	Negative
Nitrite (negative)	2+ (H)
Leukocyte esterase (negative)	500 Leu/uL (H)
Specific gravity test strip (1.005–1.030)	1.010
Urine red blood cells (0–5/HPF)	25–50
White blood cells (0–5/HPF)	>50
Urine bacteria	1+
Squamous epithelial cells (0–5/HPF)	0–2
Waxy casts (0/LPF)	10–25
Creative‐urine (measured) (30.5–125.0 mg/dL)	17.6 mg/dL (L)
Potassium, random urine (12–75 mmol/L)	83.6 mmol/L (H)
Urine culture, sterile	20,000 cfu/mL *Escherichia coli*

Abbrevations: H, high; HPF, high‐powered field; L, low; LPF, lower powered field.

In the face of presumed UTI, the infant was started on ceftriaxone. The urine culture grew *E. coli* after 24 h, and intravenous antibiotics were switched to cephalexin on hospital day 3. Renal ultrasound revealed a markedly dilated and tortuous left ureter with uroepithelial thickening and debris consistent with inflammation. The ureter appeared to extend beneath the bladder neck. Left central and peripheral caliectasis with pelviectasis was noted with uroepithelial thickening and debris. Additionally, there was circumferential bladder‐wall thickening, concerning for cystitis. Urology recommended the placement of a Foley catheter with continuation of antibiotics. After completion of an appropriate antibiotic course and clinical recovery, the infant was switched to prophylactic sulfamethoxazole/trimethoprim.

Additional assessments during the hospitalization included a bedside swallow evaluation and stool evaluation. Speech therapy noted adequate feeding readiness cues, fast feeding with endurance, and adequate intake. However, he required external pacing because of gulping, coughing, and choking. Stool‐reducing substances were negative, making food‐protein‐induced enterocolitis unlikely. The infant had an average weight gain of 55 g/day during hospitalization.

On hospital day 3, the infant’s left foot had a dusky appearance and decreased capillary refill. His left femoral arterial line was removed, and arterial Doppler ultrasound revealed a nonocclusive left external iliac thrombus. Hematology recommended treatment dosing of enoxaparin with a goal anti‐Xa level of 0.5–1 for a 12‐week course. He was discharged with good weight gain, normalized electrolytes, and no growth on subsequent urine cultures.

**Figure 1:**
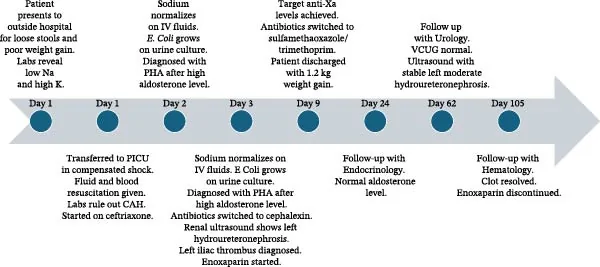
Timeline. CAH, congenital adrenal hyperplasia; PHA, pseudohypoaldosteronism; VCUG, voiding cystourethrogram.

Three weeks after discharge, a repeat aldosterone level was significantly lower at 5.1 ng/dL than the initial 315 ng/dL (5–90 ng/dL), and electrolytes remained normal. The patient was ultimately diagnosed with transient sPHA in the setting of a UTI. A follow‐up voiding cystourethrogram (VCUG) showed no vesicoureteral reflux or obstruction, but renal ultrasounds continue to show stable left moderate hydroureteronephrosis. He remains on sulfamethoxazole/trimethoprim prophylaxis. Enoxaparin was discontinued after hematology/oncology follow‐up and resolution of the thrombus. The infant’s clinical course is summarized in Figure 1. He is now at the 18th percentile for weight and growing well.

## 3. Discussion

In the kidney, aldosterone acts on the mineralocorticoid receptor to absorb sodium and excrete potassium. PHA occurs when there is an abundance of aldosterone secreted but it is unable to act on the receptor. There are three types. Type 1 is also divided into two subtypes. The autosomal recessive form is caused by inactivating mutations in the amiloride‐sensitive epithelial sodium channel (ENaC) and usually requires lifelong sodium replacement [1, 2]. The autosomal dominant form is caused by mutations in the mineralocorticoid receptor gene (*NR3C2*) and causes a less severe phenotype that resolves as a child matures [2]. Type 2 PHA (Gordon syndrome) is an autosomal recessive disorder caused by variants in the *WNK1*, *WNK4*, *Cullin3*, and *KLHL3* genes, leading to a gain‐of‐function in the thiazide‐sensitive Na–Cl cotransporter [2]. PHA type 2 presents with unexplained hyperkalemia and hypertension and is treated with thiazide diuretics [2]. Type 3 transient, acquired, or sPHA is associated with a temporary resistance of aldosterone in the setting of UTI or urinary tract malformation in infancy [1]. With all three of these PHA types, the aldosterone level is high, yet the child exhibits hyponatremia, hyperkalemia, and metabolic acidosis, indicating aldosterone resistance [3].

The pathogenesis of sPHA is not completely understood, but several hypotheses exist. Ordinarily, aldosterone activates the mineralocorticoid receptor on the tubular proximal cell of the cortical collecting duct, leading to sodium reabsorption and potassium excretion. The electrolyte shifts occur through an Na/K‐ATPase, an ENaC, and a renal medullary K+ channel once activated. When children have urinary tract malformations, there is increased pressure on the kidney and parenchymal renal damage from inflammation and bacterial endotoxins, which can downregulate the aldosterone receptor [4, 5]. This also increases cytokines and intrarenal hormones like prostaglandins and tumor necrosis factor beta 1, which can inhibit the action of aldosterone [4–6]. When there is aldosterone resistance, such as in PHA, the ENaC expression is downregulated, and the Na/K‐ATPase activity is reduced. Aldosterone levels are high in healthy young infants to maintain normal electrolytes, as the renal tubules are immature. Hence, PHA occurs more often in younger patients [5].

Most case reports published involving sPHA demonstrate infants presenting with poor weight gain, emesis, or both, as in our patient [4, 6–8]. A systematic review found that almost all cases (92%) occur in infants younger than 6 months, and the risk of sPHA in the setting of pyelonephritis significantly declines after the age of 3 months, likely due to maturation of the renal tubules [5, 9]. In a reviews of sPHA, ~90%–92% were associated with urinary tract malformations, anomalies, or both, with vesicoureteral reflux being the most common (47%) [5, 10]. Additionally, ~82% of cases occurred in males; this is thought to be related to the increased prevalence of obstructive uropathies and UTI in male infants when compared with females [5]. Abraham et al.’s [8] case series disucssed five infants who presented with hyponatremia in the setting of PHA, and all were diagnosed with a UTI. Four of these infants had congenital anomalies of the kidney [8]. In their case series, Abu Bakar et al. [11] discussed three infants who presented with hyponatremia and hyperkalemia in the setting of UTI. However, only one case mentioned testing of 17‐hydroxyprogesterone and aldosterone (normal and elevated, respectively). The authors explained that while poor fluid and nutritional intake and diarrhea may cause hyponatremia, hyperkalemia would not be observed in this clinical context, suggesting that the electrolyte abnormalities were related to the reduced activity of aldosterone [11]. Another case series detailed eight male infants with hyponatremia, hyperkalemia, and PHA; every patient had a UTI and urinary tract malformation [12]. The authors also reported that, in their review of 93 patients in the literature with PHA, 90% had urinary tract malformation and 89% had associated UTI [12]. Our patient’s underlying left hydroureteronephrosis likely predisposed him to a UTI, which is strongly correlated with sPHA.

The diagnosis of sPHA is crucial to consider in the differential diagnosis with metabolic derangements, including hyponatremia and hyperkalemia, as treating the underlying cause, including infection, usually leads to a brisk correction of the electrolytes. In review of previous cases, the infants all recovered after treatment with antibiotics for UTI [4, 6–8], intravenous hydration [4, 7, 8], sodium supplementation [4, 6–8], and/or surgical correction of the urinary tract malformation [4, 7, 8]. The patient in our case also improved with hydration and antibiotics, and he did not require sodium replacement or surgery to treat his condition. Additionally, his aldosterone normalized in a laboratory recheck 3 weeks after hospital discharge, supporting the diagnosis of sPHA. Other cases in the literature note varied time periods for the normalization of the aldosterone level—2 days [8], 4 months [8], and 11 months [4], respectively. In a diagnostic challenge, some clinicians may mistakenly diagnose the lab abnormalities of PHA as CAH, a life‐threatening condition that also presents with hyponatremia, hyperkalemia, failure to thrive, and cardiovascular shock if exhibiting symptoms of an adrenal crisis. Previous case reports detail infants treated with hydrocortisone for an incorrect diagnosis of CAH before the diagnosis of sPHA was made, which may carry risk to the patient [4, 6, 8].

Genetic testing for hereditary forms of PHA (including mutations in the mineralocorticoid receptor or ENaC) should be considered in patients with persistent or recurrent electrolyte abnormalities, poor response to standard therapy, a strong family history, or features suggestive of systemic involvement. In contrast, transient sPHA is typically characterized by the rapid resolution of biochemical abnormalities following treatment of the underlying cause. In our patient, normalization of electrolytes after antibiotic therapy and volume resuscitation without a recurrence supports a diagnosis of sPHA, and therefore, genetic testing was deferred.

The infant in our case also had the rare complication of a left external iliac thrombus. While the literature review did not find a direct connection between PHA and formation of blood clots, patients with sPHA in the setting of infection may exhibit several risk factors that predispose them to developing thrombi. In our patient, the thrombus was attributed to arterial line catheter placement, which was technically challenging at placement due to profound dehydration and poor perfusion (secondary to a systemic inflammatory state with compensated shock).

Additionally, the infant’s hemoglobin was initially low, at 7.8 g/dL. Though iron deficiency was considered because the infant was breastfeeding without an iron supplement at home, it was also thought to be a combination of critical illness and a diluted value after robust fluid resuscitation. Iron studies were not obtained. The infant’s hemoglobin normalized on day 5 of hospitalization (10.9 g/dL).

This case adds to the limited literature on transient sPHA and underscores the importance of distinguishing sPHA from congenital adrenal disorders to prevent unnecessary corticosteroid exposure. As with all single‐patient case reports, the primary limitation is limited generalizability. While the findings align with known mechanisms of sPHA, they cannot establish causality or determine the prevalence. Although the patient’s clinical and biochemical improvement supports a diagnosis of transient sPHA, more detailed characterization of family history and consideration of genetic testing could have further excluded milder hereditary forms of PHA. However, the rapid normalization of electrolytes following treatment of the underlying infection and absence of recurrence makes a hereditary etiology less likely. Additionally, serial aldosterone and renin measurements could have provided a more detailed understanding of hormonal normalization. Nevertheless, the case presented highlights a key clinical takeaway—secondary (or transient) PHA should be considered in infants presenting with hyponatremia and hyperkalemia, particularly in the context of a UTI.

## Funding

The authors did not receive any funding for this article.

## Ethics Statement

The authors declare that the research presented in this manuscript adheres to the ethical principles outlined by the Nemours Case Report Policy. Per institutional policy, a Nemours Associate may write and submit a case report of one or two individuals obtained from existing information without prior IRB review and approval. All procedures involving human participants were conducted in accordance with the ethical standards of Nemours Children’s Health and the Declaration of Helsinki (1964), as revised in 2013.

## Consent

The patient’s mother gave verbal and written consent for this case to be published. Consent form is available upon request.

## Conflicts of Interest

The authors declare no conflicts of interest.

## Data Availability

Data sharing is not applicable to this article, as no datasets were generated or analyzed during the current study.
